# Clinical utility of circulating tumor DNA profiling in detecting targetable fusions in non-small cell lung cancer

**DOI:** 10.3389/fonc.2024.1463341

**Published:** 2024-10-24

**Authors:** Young-gon Kim, Boram Lee, Changhee Ha, Cheonghwa Lee, Hyun Ae Jung, Jong-Mu Sun, Se-Hoon Lee, Myung-Ju Ahn, Yoon-La Choi, Sehhoon Park, Jong-Won Kim

**Affiliations:** ^1^ Department of Laboratory Medicine and Genetics, Samsung Medical Center, Sungkyunkwan University School of Medicine, Seoul, Republic of Korea; ^2^ Department of Pathology and Translational Genomics, Samsung Medical Center, Sungkyunkwan University School of Medicine, Seoul, Republic of Korea; ^3^ Division of Hematology-Oncology, Department of Medicine, Samsung Medical Center, Sungkyunkwan University School of Medicine, Seoul, Republic of Korea

**Keywords:** circulating-tumor DNA, ctDNA, gene fusion, non-small cell lung cancer, comprehensive genomic profiling

## Abstract

**Introduction:**

Numerous studies have suggested high concordance between tissue and circulating tumor DNA (ctDNA) comprehensive genomic profiling (CGP) tests but only few of them focused on fusions. In addition, atypical breakpoints occasionally detected from DNA-based fusion detection make interpretation difficult, and their clinical significance remains unclear. This study evaluated the clinical utility of ctDNA CGP for fusion detection.

**Methods:**

The results of ctDNA CGP tests performed on patients with stage IV non-small cell lung cancer during routine clinical care were retrospectively reviewed. The concordance between ctDNA CGP and combined tissue test results was analyzed using CGP, immunohistochemistry, fluorescence *in situ* hybridization, and reverse transcription polymerase chain reaction. The clinical significance of fusions detected by ctDNA CGP, including those with atypical breakpoints at the DNA level, was assessed.

**Results:**

In total, 264 patients were tested with ctDNA CGP. Fusions were detected in 27 patients (10.2%), and the fusion drivers were *RET* (n=12, 4.6%), *ALK* (n=9, 3.4%), *ROS1* (n=4, 1.5%), and *FGFR2* (n=2, 0.8%). The overall prevalence of fusion in tissue CGP was comparable to that in ctDNA CGP. A total of 371 ctDNA-tissue test pairs were available, and the overall positive and negative percent agreement rates were 92.9% (13/14) and 100.0% (357/357), respectively. One *ALK* IHC-positive and ctDNA CGP-negative case did not respond to *ALK*-targeted therapy. Response to targeted therapy was assessed in 16 patients, and a partial response was achieved in all patients, including four with atypical breakpoints.

**Conclusion:**

Fusion detection using ctDNA CGP showed high concordance with tissue tests and accuracy in predicting therapeutic responses in patients with non-small cell lung cancer. ctDNA CGP may provide an important diagnostic tool for fusion detection.

## 1 Introduction

Owing to the various genetic alterations that can guide therapy selection, molecular testing of tumor specimen has become essential in non-small cell lung cancer (NSCLC) treatment (1, 2). Among the different types of genetic alterations, fusions or rearrangements resulting in the activation of kinases such as ALK, ROS1, and RET are preferred treatment targets based on their superior efficacy and tolerability when treated with appropriate target agents (3–7). In addition to the conventional methods for fusion detection such as fluorescence *in situ* hybridization (FISH) and reverse transcription polymerase chain reaction (RT-PCR), high expression of immunohistochemistry (IHC) has demonstrated high concordance rate with genomic alteration especially in *ALK* and *ROS1* fusion. Recently, next-generation sequencing (NGS)-based comprehensive genomic profiling (CGP) tests have been increasingly adopted in clinical practice, and NGS of DNA and/or RNA has become the diagnostic method of choice owing to its superior accuracy and versatility (5, 8). CGP tests are particularly useful when multiple molecular targets need to be evaluated from a limited amount of specimen by detecting various types of genetic alterations, including fusions and amplifications.

Until recently, the tissue CGP test was considered the gold standard method of diagnosis because of its high accuracy and low false negative rates. However, owing to the clinical limitation in the acquisition of tissue samples and difficulties in repetitive approach due to the high invasiveness especially in lung cancer, plasma circulating tumor DNA (ctDNA) has emerged as an alternative to tissue specimen as input material for CGP (1, 9). In ctDNA CGP, fraction of tumor-derived DNA in circulation (tumor fraction, TF) is a key parameter that affects assay sensitivity (10). To detect variants with low allele fraction, as low as 0.1–0.5%, ctDNA CGP is normally performed with ultra-high sequencing volume targeting higher depth-of-coverage (DOC) than in tissue CGP (11). As a result, given that TF is sufficiently high, variants can be reliably detected using ctDNA CGP. The sensitivity of ctDNA CGP in detecting variants, including fusions, is nearly 100% when TF is above 10% (10). Large-scale retrospective review of ctDNA CGP tests performed as routine clinical testing revealed that the prevalence of fusions involving major drivers such as *ALK*, *ROS1*, *RET*, and *FGFR2* detected by ctDNA CGP was comparable to that detected using tissue CGP when TF was higher than 1% (12, 13).

In addition to TF, the major challenge in detecting fusions using plasma ctDNA is the lack of utilization of RNA. Fusions are subdivided into direct and composite, depending on the number of rearrangement events (14). RNA sequencing is considered superior to DNA sequencing in identifying fusions because RNA reflects the final product of composite fusion events. In contrast, DNA sequencing captures a snapshot of the fusion composite formation process. Interpreting gene fusions detected from tissue DNA sequencing can be challenging, especially when atypical configurations are involved, such as fusions with intergenic regions, fusions in the antisense direction, or fusions where only the reciprocal fusion is detected (15, 16). Although some of these fusions identified through DNA sequencing form canonical fusions at RNA level, others resulted in nonproductive rearrangements that do not produce fusion transcripts or proteins (16). For example, majority of the RET fusions observed to have out-of-frame configurations in DNA sequencing turned out to be in-frame in RNA sequencing (17). Owing to this indirect nature of DNA sequencing in detecting fusions, in the analysis of tissue, RNA has been widely used for fusion detection in addition to DNA (18–22). However, unlike tissue RNA, the utilization of circulating tumor RNA (ctRNA) is still under investigation (13).

This study aimed to validate the clinical utility of fusion detection using plasma ctDNA CGP. ctDNA CGP tests performed during routine clinical care of patients with NSCLC, along with tissue test results and clinical responses, were retrospectively reviewed.

## 2 Methods

### 2.1 Participants

Patients with stage IV NSCLC and tested with ctDNA CGP in routine clinical practice at the Samsung Medical Center (SMC) from December 2022 to April 2024 were included. The results of ctDNA CGP tests were retrospectively reviewed, and for patients with ctDNA CGP test results at multiple time points, the first test was used to prioritize pretreatment specimens with a higher tumor burden. The tissue test results were reviewed for concordance. For patients who underwent targeted therapy based on the fusions detected from ctDNA CGP, including those with atypical configurations, the responses were reviewed from the electronic medical records. This study was approved by the Institutional Review Board (IRB) of the SMC, Seoul, Korea (approval numbers 2024-01-133 and 2024-05-063).

### 2.2 CGP of ctDNA

The TruSight Oncology 500 (TSO500) ctDNA assay (Illumina, San Diego, CA, USA), 1.94 Mb panel covering 523 genes, was used for ctDNA CGP (**Supplementary Table S1**). The TSO500 ctDNA assay provides hybrid capture-based fusion detection, similar to most other ctDNA CGP assays. Whole blood was collected from each patient into two Cell-Free DNA Collection Tubes (Roche, Basel, Switzerland). DNA was extracted manually from 8 mL of plasma with a QIAamp DSP Circulating NA Kit (Qiagen, Hilden, Germany) according to the manufacturer’s instructions. Libraries were prepared using 30 ng of input DNA, according to the TSO500 ctDNA assay protocol provided by the manufacturer. Subsequently, the library was enriched for 523 genes using a pool of target-specific oligos during two rounds of hybridization and target capture, followed by a final library amplification and cleanup. Quality control procedures before and after library preparation were performed using a Qubit (Thermo Fisher Scientific, Waltham, MA, USA) and a Cell-free DNA ScreenTape Assay (Agilent, Santa Clara, CA, USA). Sequencing was performed using an Illumina NovaSeq 6000 instrument with a read length of 2 × 150 bp and a targeted sequencing depth of 800 million reads (120 Gb) per sample. Bioinformatic analyses were performed using the DRAGEN TSO500 ctDNA Analysis Software version 1.1.0 on an Illumina DRAGEN Server, according to the user guide provided by the manufacturer. Within the software, fusion calling step used the Manta fusion caller, considering candidate fusions with at least 3 unique supporting reads, one of which must be a split read (a single read crossing the fusion breakpoint).

### 2.3 TF estimation

TF can be quantified either using aneuploidy- or maximum somatic allele frequency (MSAF)-based methods (12, 23). As the aneuploidy-based method is applicable only to cases with relatively high TF, at least 3% according to a landmark research, (24) MSAF-based methods have been used to complement aneuploidy-based methods for cases with low TF (12, 13, 23). In this study, to differentiate between cases with sufficient TF [≥ 1% following the previous studies (12, 13)] and those with limited TF (< 1%), MSAF of 0.5%, which is equivalent to TF of 1%, was used as the threshold. MSAF was determined as the highest VAF observed from the list of somatic variants generated by the TSO500 DRAGEN software for tumor mutation burden (TMB) calculation. The TMB variant list was generated by filtering out potential germline variants and variants of genes commonly associated with clonal hematopoiesis from all the variants detected in the coding region.

### 2.4 Acquisition of tissue-based test results

Tissue-based molecular testing results, including ALK IHC, *ALK* FISH, ROS1 IHC, *ROS1* RT-PCR, and tissue CGP, were reviewed from electronic medical records. When multiple tissue test items were evaluated for the same fusion target, combined tissue results were used. When a discrepancy was observed between the results of IHC and RT-PCR, the results from the latter were used as true values, considering the higher accuracy of RT-PCR compared to that of IHC (5, 8).

To compare the overall prevalence rate of fusion between tissue and ctDNA CGP, the results of tissue CGP performed on patients with NSCLC in SMC during the study period, from December 2022 to April 2024, were used. Tissue CGP tests were performed using the tissue version of the TSO500 assay, and fusion detection was performed by hybridization capture-based enrichment of target regions using tissue RNA. Although the fusion drivers targeted by the two assays were different (55 genes in the tissue and 23 genes in the ctDNA versions, **Supplementary Table S1**), the most important fusion drivers in NSCLC, namely ALK, ROS1, and RET, were commonly targeted in both assays, and a comparison was performed for these three genes. For cases in which fusion was detected from both ctDNA and tissue CGP, their raw data (BAM files) were compared to observe the difference in fusion configurations in DNA and RNA.

### 2.5 Statistical analysis

Concordance between the combined tissue results and ctDNA CGP was analyzed in terms of positive percent agreement (PPA) and negative percent agreement (NPA) using combined tissue results as a reference. The positive predictive value (PPV) of ctDNA CGP was also assessed using combined tissue results. Fisher’s exact test was used to compare categorical variables between groups, and a binomial test was used to compare proportions. Statistical significance was set at P < 0.05. Statistical analyses were performed using the R software (version 4.2.2).

## 3 Results

### 3.1 Fusions detected from ctDNA CGP

**Figure 1:**
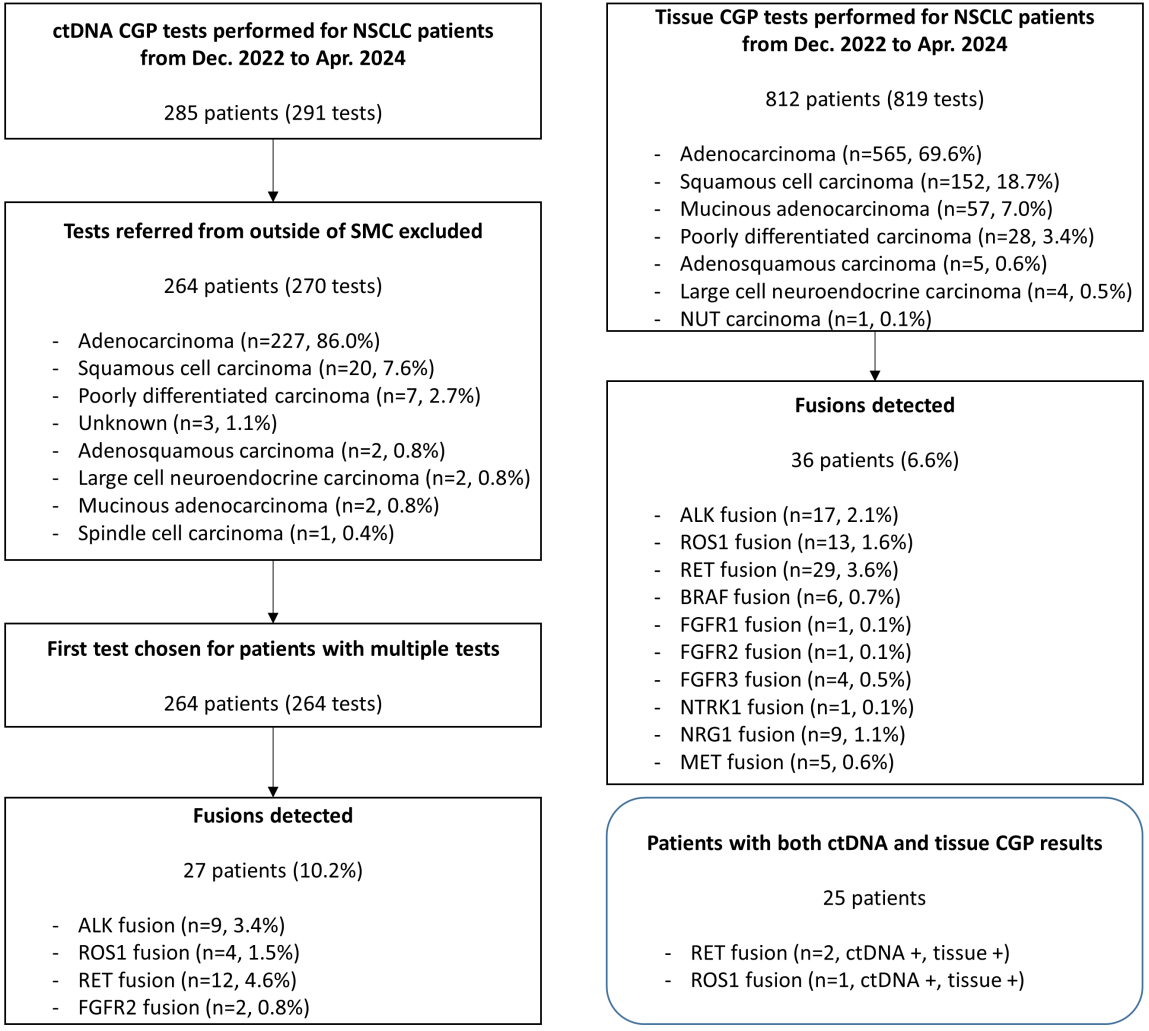
Flowchart of study inclusion and summary of the detected fusions. During the study period, 291 ctDNA CGP tests were performed on samples from 285 patients with NSCLC. After exclusion of tests referred from outside of SMC, 264 patients were finally included, and fusions were detected from 27 cases (10.2%). Tissue CGP was performed in 812 patients with NSCLC during the same period and fusion was detected from 86 patients (10.6%). Twenty five patients had both ctDNA and tissue CGP results and fusions were detected from three of them, commonly from ctDNA and tissue CGP. ctDNA, circulating tumor; CGP, comprehensive genomic profiling; MSAF, maximum somatic allele frequency; NSCLC, non-small cell lung cancer; SMC, Samsung Medical Center.

A total of 264 patients were tested for ctDNA CGP during the study period, and their histological subtypes included adenocarcinoma (n=227, 86.0%), squamous cell carcinoma (n=20, 7.6%), and poorly differentiated carcinoma (n=7, 2.7%) (**Figure 1**). Fusions were detected in 27 patients (10.2%), including *RET* (n=12; 4.6%), *ALK* (n=9; 3.4%), *ROS1* (n=4; 1.5%), and *FGFR2* (n=2; 0.8%) (**Table 1**). All *ALK* and *ROS1* fusions were detected in adenocarcinomas, and *RET* fusions were detected mostly in adenocarcinomas (11/12, 91.7%), except in one case of poorly differentiated carcinoma. *FGFR2* fusions were detected in one case each of adenocarcinoma and squamous cell carcinoma.

**Table 1:** Fusions variants detected from ctDNA CGP.

Case ID	Tissue type	Driver (Exon)	Partner (Exon)	VAF (%)	MSAF (%)	Fusion configuration	Reciprocal rearrangement	Tissue result	Fusion- targeted Treatment^*^	Clinical response
SMC002	ADC	ALK (20)	EML4 (13)	0.14	0.99	Canonical	Found	IHC(+) FISH(+)	Lorlatinib	PR
SMC086	ADC	ALK (20)	EML4 (6)	0.08	0.72	Canonical	Not found	IHC(+)	Alectinib	Not available
SMC113	ADC	ALK (20)	EML4 (13)	0.11	0.11	Canonical	Not found	IHC(+)	Not done	Not available
SMC143	ADC	ALK (20)	EML4 (6)	0.92	5.28	Canonical	Found	IHC(+)	Brigatinib	PR
SMC145	ADC	ALK (20)	EML4 (6)	2.83	3.46	Canonical	Not found	Not assessable	Brigatinib	PR
SMC193	ADC	ALK (20)	ALK (12)	2.4	4.56	Atypical breakpoint	Not found	IHC(+)	Lorlatinib	PR
SMC198	ADC	ALK (20)	DYNC1I1 (7)	0.23	1.45	Atypical breakpoint	Found	IHC(+)	Alectinib	PR
SMC255	ADC	ALK (20)	EML4 (13)	1.17	1.15	Canonical	Not found	IHC(+)	Alectinib	PR
SMC259	ADC	ALK (20)	EML4 (13)	0.51	2.6	Canonical	Found	IHC(+)	Lorlatinib	PR
SMC114	ADC	ROS1 (34)	EZR (10)	0.09	4.19	Canonical	Not found	Not available	Not done	Not available
SMC182	ADC	ROS1 (31)	SDC4 (2)	0.2	0.48	Atypical breakpoint	Not found	IHC(+) RT-PCR(+) NGS(+)	Crizotinib	PR
SMC222	ADC	ROS1 (32)	CD74 (7)	2.13	2.86	Canonical	Not found	IHC(+) RT-PCR(+)	Crizotinib	PR
SMC245	ADC	ROS1 (32)	EZR (10)	3.95	17.29	Canonical	Not found	IHC(+) RT-PCR(+)	Entrectinib	PR
SMC020	ADC	RET (12)	KIF5B (15)	2.6	2.6	Canonical	Found	Not available	Selpercatinib	PR
SMC028	ADC	RET (11)	DNAJC1 (1, 2)	1.59	6.58	Atypical breakpoint	Found	Not available	Selpercatinib	PR
SMC031	ADC	RET (12)	KIF5B (23)	0.18	0.89	Canonical	Found	Not available	Not done	Not available
SMC068	ADC	RET (10)	KIF5B (24)	0.41	1.18	Canonical	Found	NGS(+)	Selpercatinib	PR
SMC084	ADC	RET (12)	KIF5B (15)	0.13	0.49	Canonical	Found	Not available	Not done	Not available
SMC123	ADC	RET (12)	KIF5B (15)	0.27	0.59	Canonical	Not found	NGS(+)	Not done	Not available
SMC140	ADC	RET (12)	KIF5B (15)	11	12.88	Canonical	Not found	Not available	Selpercatinib	PR
SMC163	ADC	RET (12)	CCDC6 (1)	0.93	2.7	Canonical	Found	Not available	Not done	Not available
SMC181	ADC	RET (11)	KIF5B (16)	0.29	1.03	Atypical breakpoint	Found	Not available	Not done	Not available
SMC183	ADC	RET (12)	KIF5B (23)	0.47	1.29	Canonical	Not found	Not available	Selpercatinib	PR
SMC230	ADC	RET (12)	KIF5B (15)	2.6	4.95	Canonical	Found	Not available	Not done	Not available
SMC257	PDC	RET (12)	KIF5B (15)	19.6	33.72	Canonical	Not found	Not available	Selpercatinib	PR
SMC171	SCC	FGFR2 (17)	GRID1 (9)	0.36	1.07	Atypical breakpoint	Not found	Not available	Not done	Not available
SMC226	ADC	FGFR2 (17)	TACC2 (2)	6.76	34.19	Canonical	Not found	Not available	Not done	Not available

^*^The first fusion-targeted therapy after the result of ctDNA CGP result.

ADC, adenocarcinoma; SCC, squamous cell carcinoma; PDC, poorly differentiated carcinoma; IHC, immunohistochemistry; FISH, fluorescence in-situ hibridization; NGS, next-generation sequencing; PR, partial response; NA, not assessed; NT, not treated.

**Figure 2:**
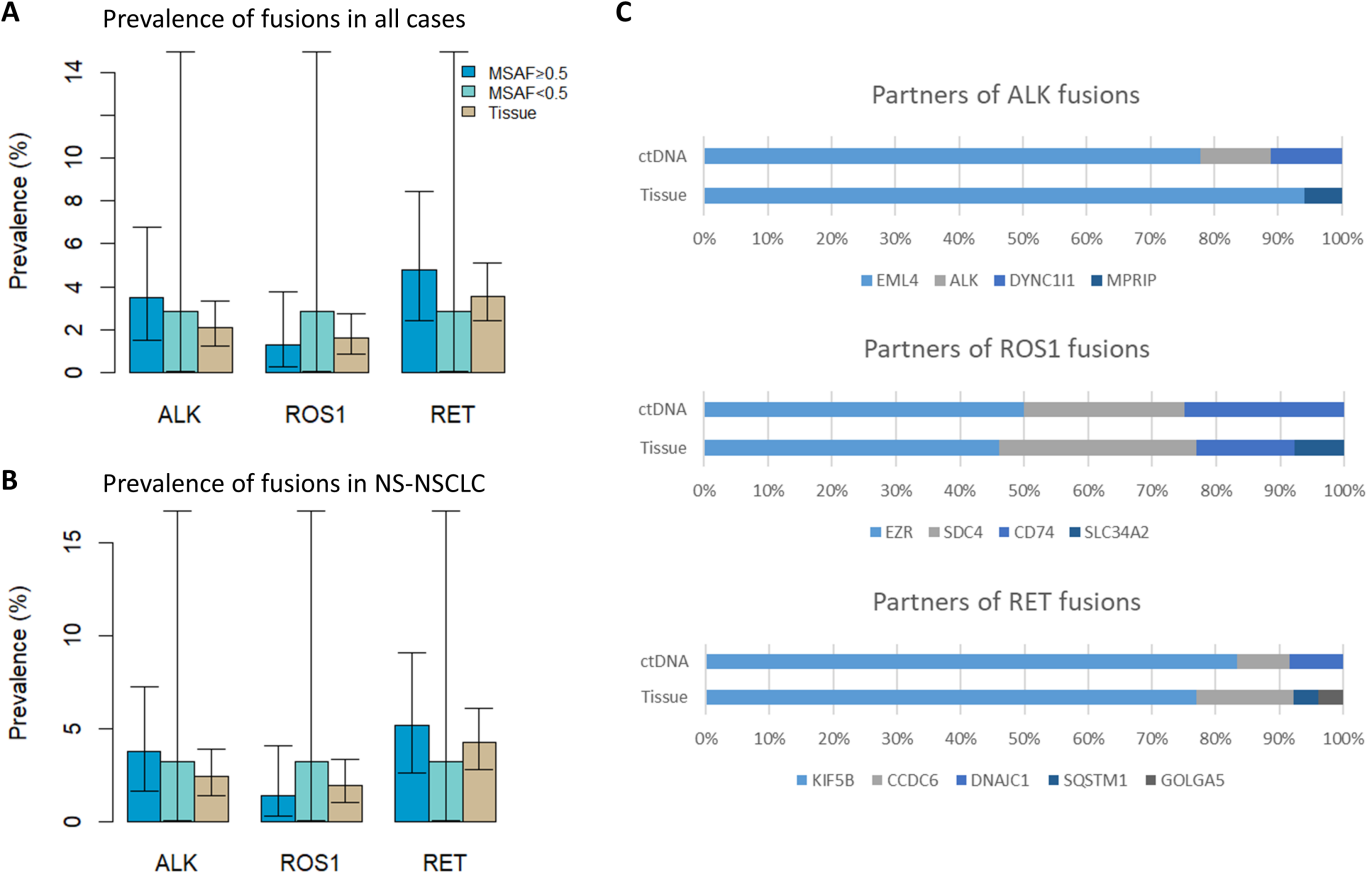
Detection of fusions from ctDNA and tissue. **(A)** Prevalence of fusions from ctDNA (MSAF ≥ 0.5 and <0.5 groups) and tissue (all cases) **(B)** Prevalence of fusions when only NS-NSCLC cases were considered **(C)** Distribution of fusion partners. ctDNA, circulating tumor; MSAF, maximum somatic allele frequency; NS-NSCLC, non-squamous non-small cell lung cancer.

The prevalence rate of *ALK*, *ROS1*, and *RET* fusions were compared between ctDNA CGP (MSAF ≥ 0.5 group and MSAF < 0.5 group) and tissue CGP (**Figure 2**). As tissue CGP had a higher proportion of squamous cell carcinoma, in which fusions were rarely detected, the prevalence rate was also derived from non-squamous (NS)-NSCLC subsets (**Figure 2B**). However, the significant difference in the fusion prevalence was not observed across groups in all fusion drivers.

**Figure 3:**
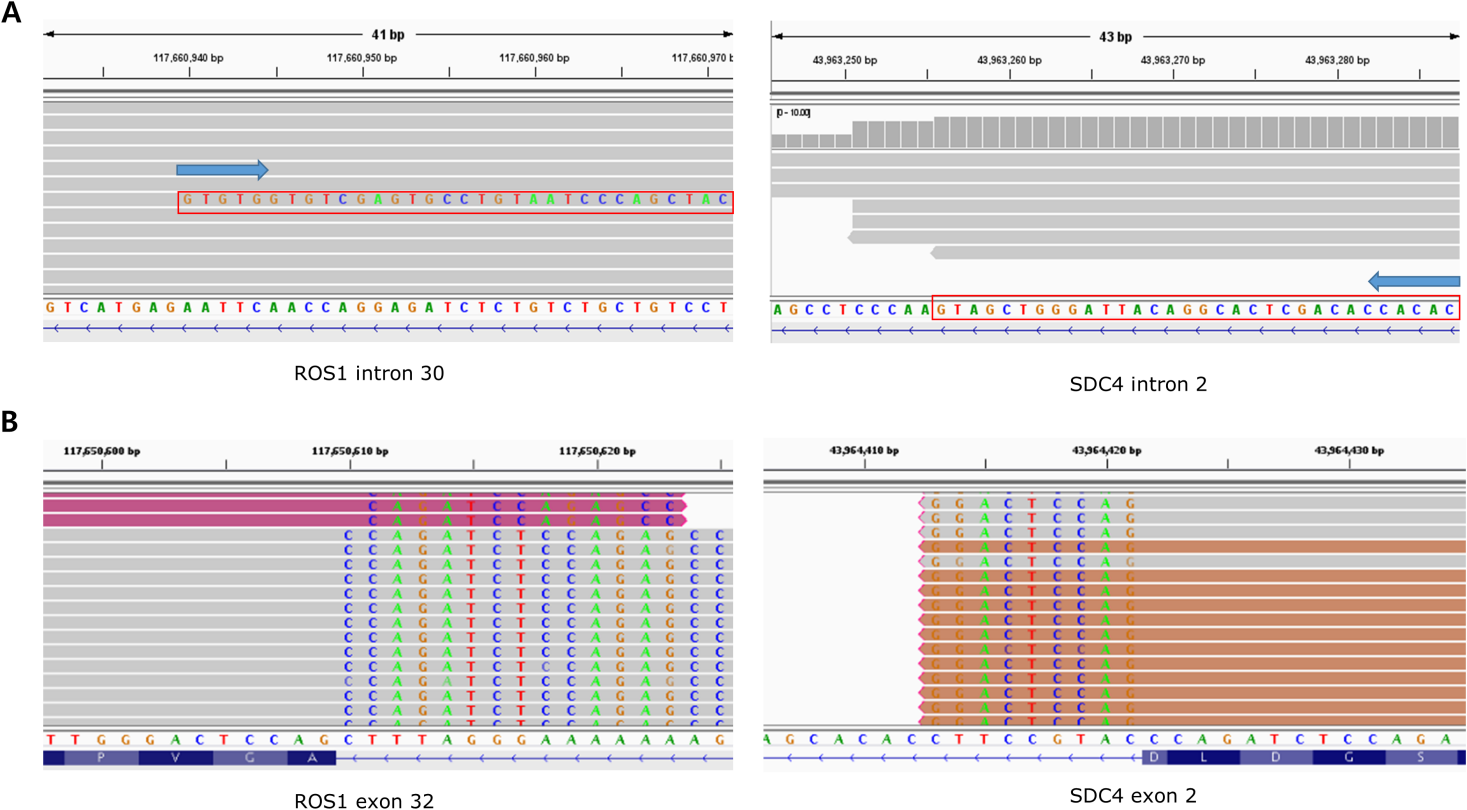
Comparison of BAM file findings of a fusion detected from SMC182 between ctDNA and tissue RNA. **(A)** From ctDNA, downstream part of ROS1 intron 30 was fused to SDC4 intron 2. However, the downstream part, rather than upstream part, of SDC4 intron 2 constituted the fusion. **(B)** From tissue RNA, ROS1 exon 32 was fused to SDC4 exon 2, which was in-frame.

**Figure 4:**
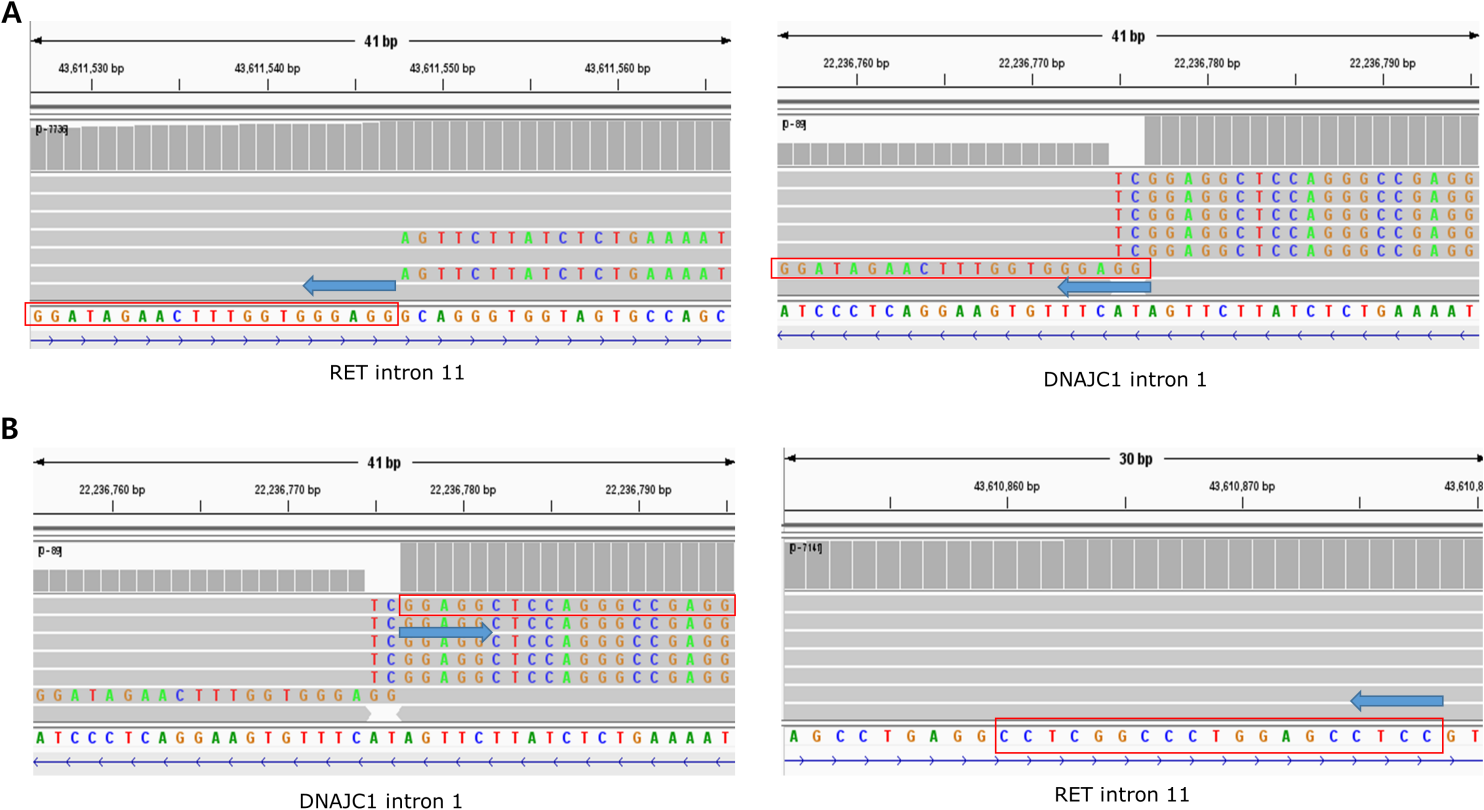
A fusion of reversed partner gene direction detected from ctDNA of SMC028. **(A)** Upstream part of DNAJC1 and upstream part of RET are fused. **(B)** The downstream part of DNAJC1, rather than upstream part, is fused to the upstream part of the RET.

The distribution of fusion partners is shown in **Figure 2C**. The majority of fusion partners for *ALK* were *EML4* in ctDNA CGP (7/9, 77.8%), while two cases had atypical configuration, and the exact partners forming in-frame fusions were not detected (SMC193 and SMC198, as shown in **Supplementary Figure S1, S2**). The fusion partners of *ROS1* were *EZR* (2/4, 50.0%), *CD74*(1/4, 25.0%), and *SDC4*(1/4, 25.0%), with the breakpoint observed in *SDC4* being non-canonical (SMC182; **Figure 3**). Fusion partners of *RET* were *KIF5B* (10/12, 83.3%), *CCDC6* (1/12, 8.3%), and *DNAJC1* (1/12, 8.3%), with two cases showing *RET* fusions involving only the 5′ region of *RET* (SMC028 and SMC181, shown in **Figure 4**; **Supplementary Figure S3**). Breakpoints of *FGFR2* commonly involved the known hotspot of *FGFR2* intron 18, and the partners were *GRID1* and *TACC2* (**Table 1**). Although *TACC2* is a common fusion partner of *FGFR2*, *GRID1* has not yet been reported.

### 3.2 Concordance between tissue and ctDNA CGP

Among the 264 cases available for ctDNA CGP, the number of cases in which combined tissue test results were available was 229 for *ALK*, 92 for *ROS1*, 25 for *RET*, and 25 for *FGFR2* fusion (**Table 2**). When all four genes were considered together, 371 ctDNA-tissue test pairs were available, and the overall PPA and NPA were 92.9% (13/14) and 100.0% (357/357), respectively. PPV was 100.0% (13/13). The only discrepant case, SMC063, was ALK IHC-positive and ctDNA CGP-negative (**Supplementary Tables S2, S3**). The MSAF of this case was 3.19% and the median unique molecular DOC was 3,075 ×, which was higher than the 2,500 × recommended by the manufacturer to ensure a 0.5% limit of detection. Based on the ALK IHC results, only approximately 10% of the tumor cells were positive, whereas the remaining tumor cells showed no immunoreactivity. The patient was treated with alectinib based on the positive ALK-IHC result. However, a PET-CT performed 2 months later revealed aggravated or newly appeared multiple hypermetabolic lesions in the right pleura, suggesting progressive disease. At the time of disease progression, ctDNA CGP was performed, which showed no alterations in ALK. T his finding raised the possibility of either a false positive IHC result or primary resistance caused by another oncogenic driver.

**Table 2:** Concordance of combined tissue tests and ctDNA CGP in fusion detection.

Combined tissue result*	ALK (N=229)**		ROS1 (N=92)		RET (N=25)		FGFR2 (N=25)		Overall (N=371)	
	ctDNA (+)	ctDNA (-)	ctDNA (+)	ctDNA (-)	ctDNA (+)	ctDNA (-)	ctDNA (+)	ctDNA (-)	ctDNA (+)	ctDNA (-)
Positive	8	1	3	0	2	0	0	0	13	1
Negative	0	220	0	89	0	23	0	25	0	357

*Combined tissue result of IHC, FISH, RT-PCR, and NGS.

**Numbers in parenthesis corresponds to the number of cases with at least one tissue test result for the fusion gene.

Both tissue and ctDNA CGP were performed in 25 cases, and fusion was detected in three cases (SMC068, SMC123, and SMC182). The driver and partner gene configurations observed in the ctDNA and RNA were identical for SMC068 and SMC123 (**Supplementary Figure S4, S5**). However, the observed breakpoint of the *ROS1* fusion in SMC182 cells differed between ctDNA and tissue RNA (**Figure 4**). In this case, while the fusion observed from ctDNA was expected to produce an out-of-frame product, a fusion between *ROS1* exon 31 and *SDC4* exon, the one observed from tissue RNA, was a fusion between *ROS1* exon 32 and *SDC4* exon 2, which was in-frame.

### 3.3 Clinical significance of fusions detected from ctDNA CGP

Among the 27 patients with fusions detected in ctDNA CGP, 17 (63.0%) underwent targeted therapy for the fusions, and a partial response was observed in all patients whose responses were assessed (16/16) (**Table 1**). Five patients (SMC020, SMC028, SMC140, SMC145, and SMC183) underwent targeted therapy based solely on the ctDNA CGP results without tissue test results, and four of them had *RET* fusions for which IHC/FISH/RT-PCR was not available.

Among the six patients whose fusion configuration was non-canonical, four were treated with targeted agents (SMC028, SMC182, SMC193, and SMC198), and all showed a partial response (**Table 1**). The objective response ratio (ORR) did not differ between patients with canonical fusions and those with atypical breakpoints (10/10 vs. 4/4, P=1.000). Specifically, SMC028 cells were treated with selpercatinib based solely on the non-canonical findings of *DNAJC1*-*RET* fusion derived from ctDNA GCP (**Figure 4**), which showed a significant response that persisted for 14 months.

Among the 16 cases in which the response was assessed, reciprocal rearrangement was observed in seven cases. The ORR was not affected by reciprocal rearrangement (7/7 vs. 9/9, P=1.000).

## 4 Discussion

Owing to the expansion of targetable biomarkers, testing for molecular markers has become an essential part of NSCLC treatment. Genetic evolutions encountered during treatment have made repeated biopsies a common practice (25). Considering the cost and complications associated with repeated biopsy procedures, ctDNA CGP is expected to play an increasingly important role (26). Although numerous studies have suggested high concordance between tissue and ctDNA CGP tests, only a few of them focused on fusions (12, 13). Owing to the perceived inferior sensitivity of ctDNA CGP and the perception that DNA is a suboptimal specimen for fusion detection compared to RNA, the clinical utility of ctDNA CGP in terms of fusion detection has not yet been fully recognized.

In this study, the results of ctDNA CGP and combined tissue tests were retrospectively analyzed, and the results were perfectly concordant, except for one case in which false positivity of immunohistochemistry (IHC) was suspected based on the response to targeted therapy. In this case, only a small proportion of the tumor cells were positive. Typically, any percentage of positive tumor cells was considered a positive result (27). However, this case suggests that a confirmatory test may be necessary only when focal positivity is observed.

The prevalence of fusions was comparable between ctDNA and tissue CGP, supporting the concordance observed between the two methods. RET fusions have been reported to occur in 1–2% of patients with NSCLC, and the prevalence was reported to be higher in the Korean population (28–31). The prevalence rate of RET fusion observed in this study from ctDNA CGP was 4.5% (12/264), which was similar to that previously reported in Korea (30). Considering the high prevalence of RET fusions in specific populations and low availability of other tests such as RET IHC, (31) CGP tests should be considered a priority, and ctDNA could be an appropriate option based on the high clinical utility observed in this study.

Although there have been reports concerning the reliability of fusions observed to have atypical configurations using DNA-based methods, (15, 16) all cases in this study responded to targeted therapy. This result is in concordance with previous studies, in which the majority of fusions detected to have atypical configurations in DNA sequencing turned out to have canonical fusions in RNA sequencing or IHC (15–17). Validation using tissue RNA or protein assays could be warranted for available cases, as suggested in previous reports (16, 17). However, considering the potential scarcity of tissue specimens from patients who have already chosen ctDNA CGP instead of tissue, a trial of targeted therapy without confirmation could be considered based on risk vs. benefit analysis. Notably, most atypical fusions confirmed to produce canonical fusions had breakpoints located at or slightly upstream of the canonical location.

A limitation of this study was the sample size, especially for MSAF < 0.5 group. In the SMC, only patients with stage IV were eligible for ctDNA CGP, following the results of the Korean Food and Drug Administration accreditation, which might have resulted in the depletion of cases with a low tumor burden. One of the limitations of ctDNA CGP is the low applicability to cases with low tumor burden. However, the number of cases in MSAF < 0.5 group was too small for the statistical analysis and consequently, the difference in the prevalence was not observed between the MSAF ≥ 0.5 and < 0.5 groups as that observed in previous studies (12, 13). Future studies with larger number of cases, including earlier stage NSCLC cases, could help reveal the true performance of ctDNA CGP in fusion detection. On the other hand, the overall number of cases was sufficient for the comparison of prevalence between ctDNA and tissue CGP, which demonstrated that ctDNA is at least comparable to tissue CGP in detecting fusions. It should also be noted that the frequency of *ALK* fusions in tissue CGP may be underestimated because ALK IHC is prioritized and positive cases may not be tested for CGP. Lastly, the timing of tissue acquisition and blood sampling for ctDNA CGP could not be aligned owing to the retrospective nature of the study. The mutational profile, including fusions, might have been affected by the treatment or clonal evolution during the gap between tissue and blood sampling, affecting the correlation observed in this study.

In conclusion, fusion detection using ctDNA CGP showed high concordance with tissue tests and accuracy in predicting the therapeutic response in patients with NSCLC. ctDNA CGP is expected to provide an important diagnostic tool for fusion detection.

## Data Availability

The original contributions presented in the study are included in the article/**Supplementary Material**, further inquiries can be directed to the corresponding author/s.
