# Supplementary material for: Clinical utility of circulating tumor DNA profiling in detecting targetable fusions in non-small cell lung cancer
Source: Front Oncol. 2024 Oct 24;14:1463341. doi: 10.3389/fonc.2024.1463341 (PMC11540554; doi:10.3389/fonc.2024.1463341)
Supplement: Supplementary file 1 [file DataSheet1.docx]

Supplementary Material


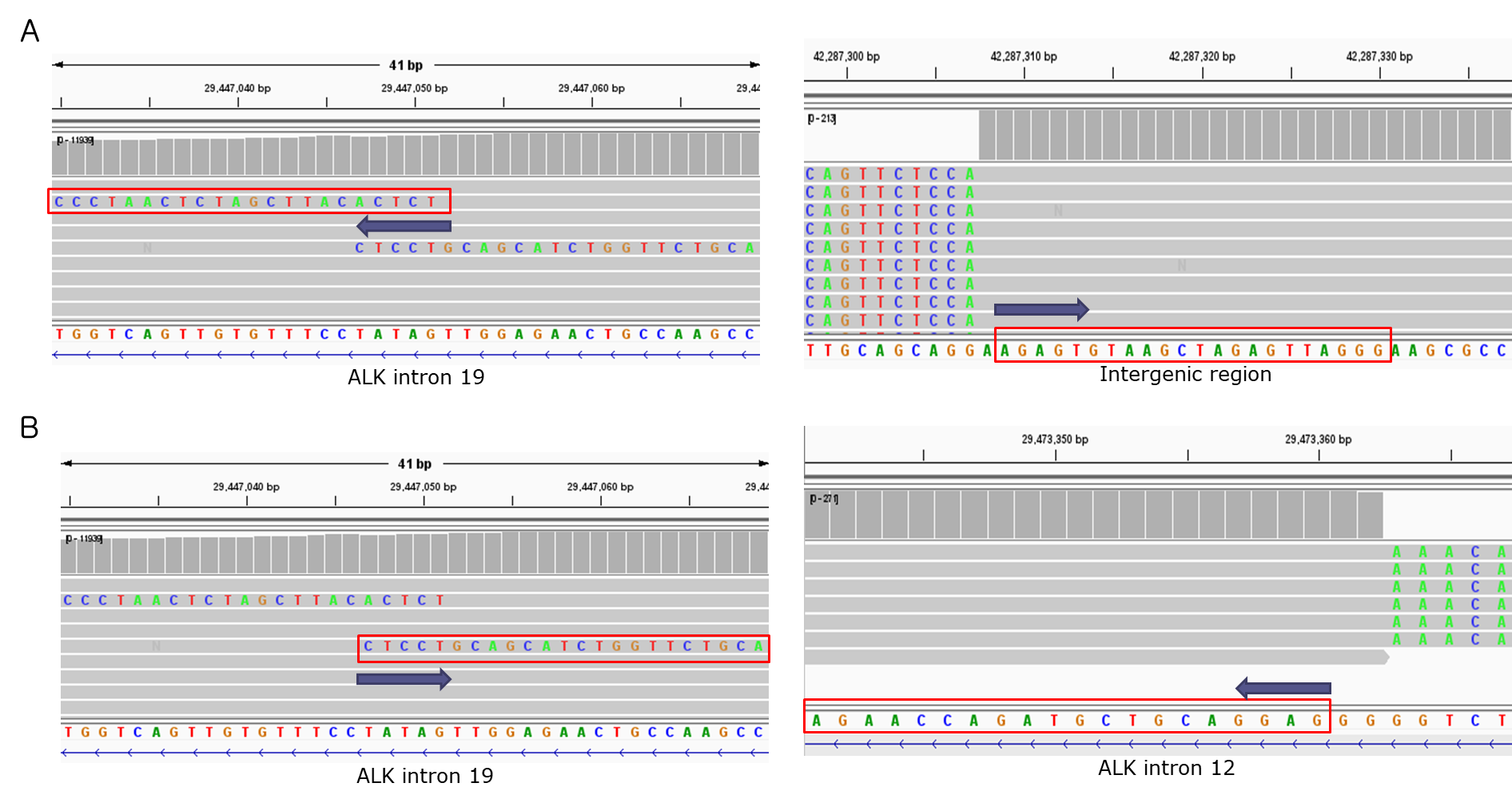


**Supplementary Figure 1.** **A case of intra-genetic fusion detected from ctDNA of SMC193**. A. Upstream part of ALK is fused to intergenic region, suggesting reciprocal rearrangement. This configuration was called by Manta. B. The downstream part of ALK intron 19 is fused to the downstream part of ALK intron 12. Considering that downstream location of ALK kinase domain, this configuration is closer to the canonical form. However, this configuration was not called by Manta.


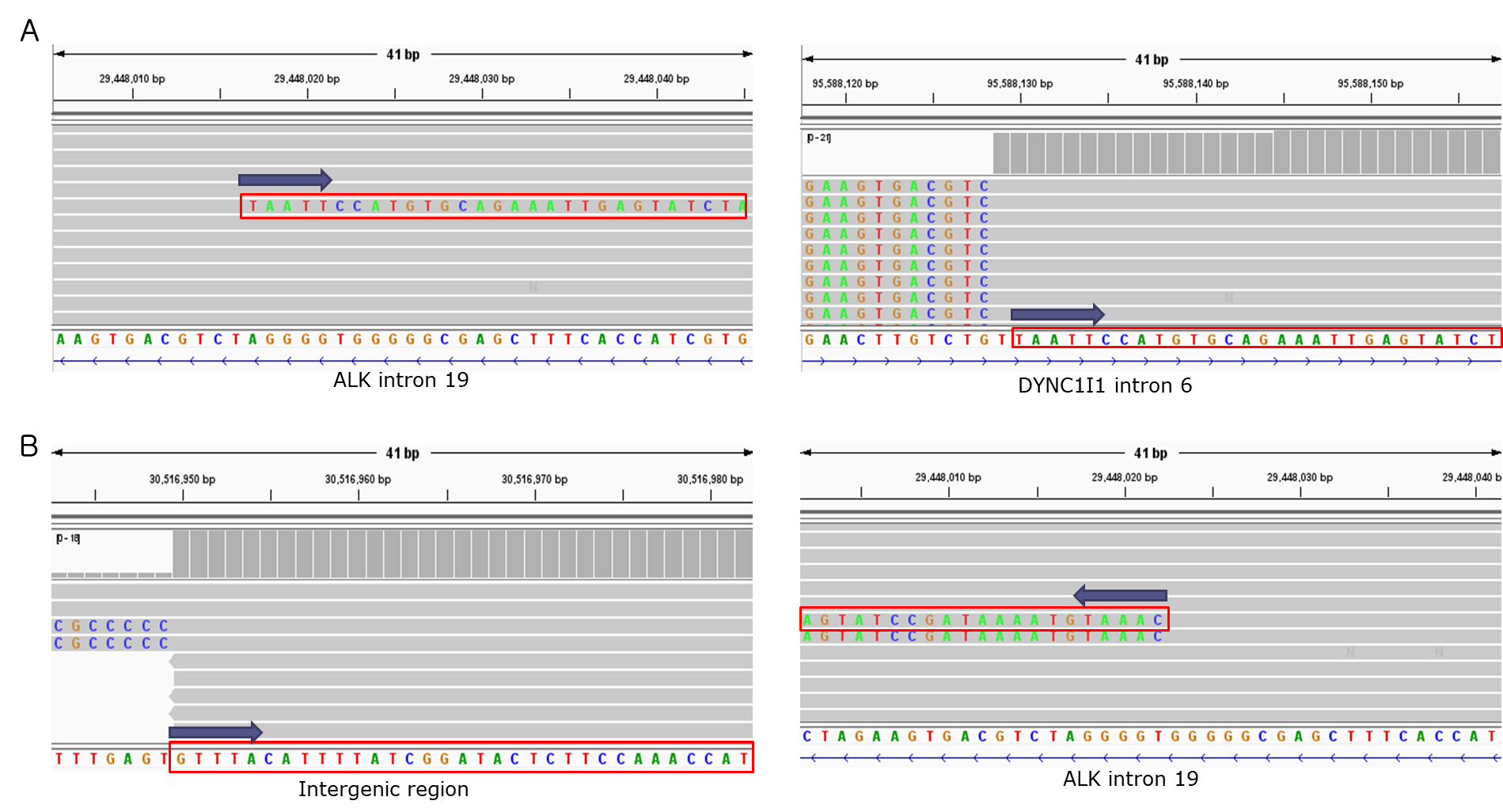


**Supplementary Figure 2.**  **A fusion of reversed partner gene direction detected from ctDNA of SMC198**. A. Downstream part of ALK intron 19 and downstream part of DYNC1I1 intron 6 are fused. B. A reciprocal rearrangement in which the upstream part of ALK and an intergenic region are fused.


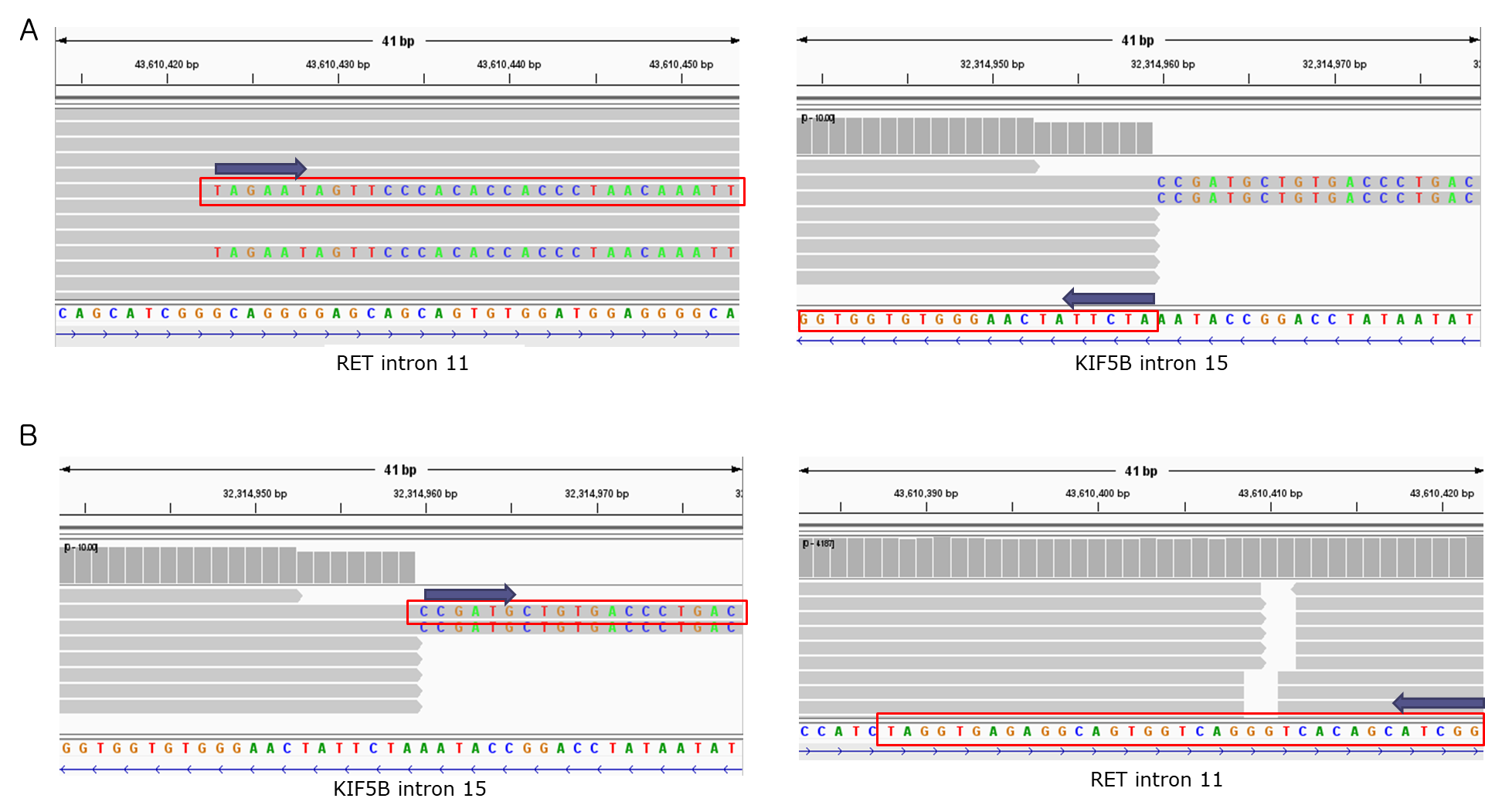


**Supplementary Figure 3.**  **Reciprocal rearrangement detected from ctDNA of SMC181.** In both A and B, downstream region of KIF5B is fused to the upstream region of RET. Findings compatible with canonical configuration was not found and the patient was not treated with RET inhibitors.


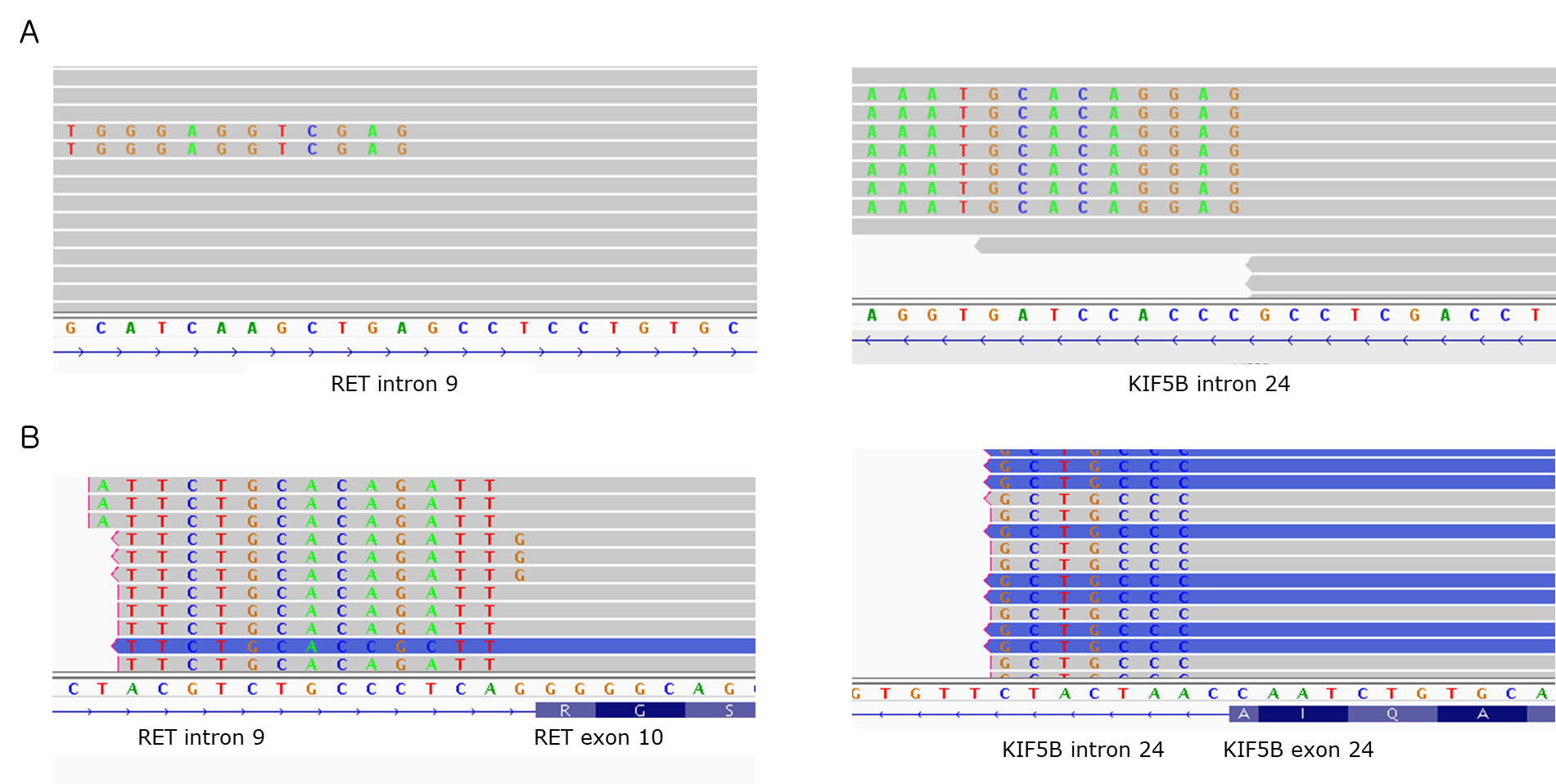


**Supplementary Figure 4.**  **Comparison of observed RET fusion breakpoints between ctDNA and RNA (SMC068).** A. From ctDNA, fusion of RET exon 10 and KIF5B exon 24 is expected. B. Tissue RNA sequencing revealed the fusion between RET exon 10 and KIF5B exon 24.


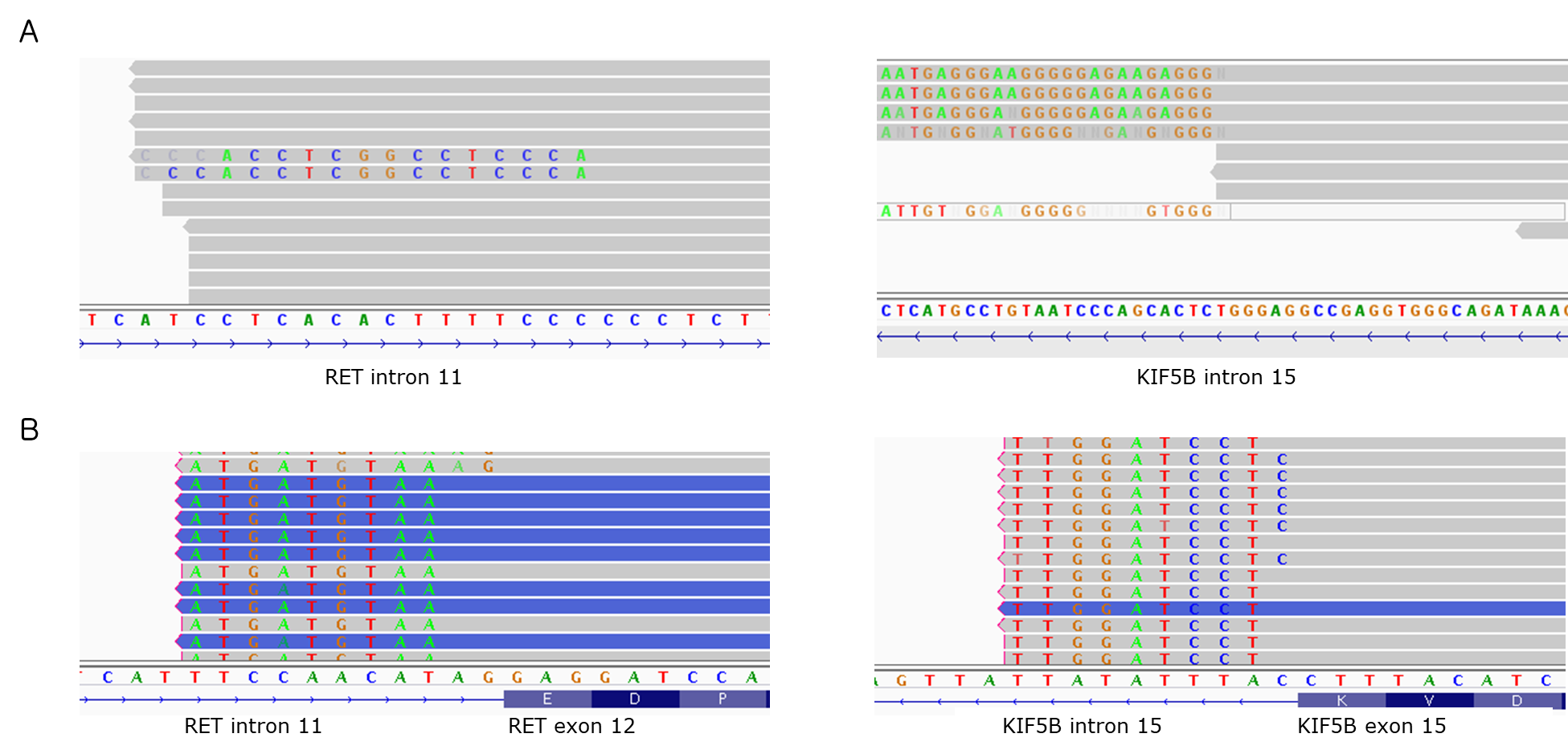


**Supplementary Figure 5.**  **Comparison of observed RET fusion breakpoints between ctDNA and RNA (SMC123).** A. From ctDNA, fusion of RET exon 12 and KIF5B exon 15 is expected. B. Tissue RNA sequencing revealed the fusion between RET exon 12 and KIF5B exon 15
